# *In situ *cognate interactions between T-follicular helper cells and B cells characterize severe tubulointerstitial inflammation in human lupus nephritis

**DOI:** 10.1186/ar3959

**Published:** 2012-09-27

**Authors:** MR Clark, M Giger, Y Jiang, V Liarski

**Affiliations:** 1 University of Chicago, IL, USA

## Background

In human lupus nephritis, the severity of tubulointerstitial inflammation on biopsy correlates with the risk of subsequent progression to renal failure [1]. Tubulointerstitial inflammation, and not glomerular inflammation, is associated with *in situ *clonal selection [2] and expansion of B cells reactive with antigens associated with inflammation. Certainly antigen recognition by the B-cell antigen receptor provides signals necessary for B-cell selection. However, additional second signals, derived from antigen-specific T cells or pattern recognition receptors, are required for B-cell proliferation. Therefore, we hypothesized that *in situ *B cells were receiving help from *in situ *T cells.

## Methods and results

Consistent with our hypothesis, using four and five color confocal microscopy we observed CD4^+^ICOS^+^PD-1^+ ^T cells in close apposition with CD20^+ ^B cells. To quantitate the relationship between these presumptive T_FH _cells and B cells, we developed novel, automated and therefore unbiased algorithms (using Python/Linux) to define the location of cells expressing multiple surface markers and then to assess the shortest distances between the same or different cell types. These analyses revealed that B cells or T_FH _cells were never in close approximation with each other. In contrast, an average of 42% of T_FH _cells in renal biopsies from lupus patients were in very close contact with B cells. Three-dimensional imagining of these conjugates revealed that closely opposed T_FH _and B cells had formed atypical supramolecular activation complexes containing polarized TCR, LFA-1, MHC class II and ICAM-1 molecules. This suggests that the T_FH _and B cells in these conjugates are antigenically related. The difference between the observed T:B conjugate rate, and that predicted to arise by chance, was highly significant at up to *P *= 10^-42^. Finally, qPCR of mRNA captured from *in situ *ICOS^+ ^T cells revealed that they had expression profiles consistent with them being *bona fide *T_FH _cells.

## Conclusion

These data demonstrate that T_FH _cells are probably contributing to *in situ *humoral immune responses in lupus nephritis. Furthermore, our results indicate that quantitative imaging can be used to reveal important cell:cell interactions in human tissue.
